# Reactive Oxygen Species and Redox Signaling in Chronic Kidney Disease

**DOI:** 10.3390/cells9061342

**Published:** 2020-05-28

**Authors:** Maria V. Irazabal, Vicente E. Torres

**Affiliations:** 1 Department of Internal Medicine, Division of Nephrology and Hypertension, Mayo Clinic, 200 First Street, Rochester, MN 55905, USA; torres.vicente@mayo.edu; 2 Mayo Translational PKD Center, Mayo Clinic, Rochester, MN 55905, USA

**Keywords:** chronic kidney disease, reactive oxygen species, oxidative stress, mitochondria, NADPH oxidases, nuclear factor erythroid 2–related factor 2 (Nrf2)

## Abstract

Chronic kidney disease (CKD) remains a worldwide public health problem associated with serious complications and increased mortality rates. Accumulating evidence indicates that elevated intracellular levels of reactive oxygen species (ROS) play a major role in the pathogenesis of CKD. Increased intracellular levels of ROS can lead to oxidation of lipids, DNA, and proteins, contributing to cellular damage. On the other hand, ROS are also important secondary messengers in cellular signaling. Consequently, normal kidney cell function relies on the “right” amount of ROS. Mitochondria and NADPH oxidases represent major sources of ROS in the kidney, but renal antioxidant systems, such as superoxide dismutase, catalase, or glutathione peroxidase counterbalance ROS-mediated injury. This review discusses the main sources of ROS and antioxidant systems in the kidney, and redox signaling pathways leading to inflammation and fibrosis, which result in abnormal kidney function and CKD progression. We further discuss the important role of the nuclear factor erythroid 2-related factor 2 (Nrf2) in regulating antioxidant responses, and other mechanisms of redox signaling.

## 1. Introduction

Chronic kidney diseases (CKD) have multiple causes, which share the common feature of repetitive cycles of injury to the glomerular or tubular epithelium, associated with changes in cellular proliferation and repair, as well as activation of inflammatory processes, fibroblasts, and fibrosis, thereby resulting in nephron loss, decline in renal function and ultimately end-stage kidney disease (ESKD). Oxidative stress has been shown to underlie many of these processes, playing a critical role in renal damage and offering a potential target for therapeutic intervention.

Oxidative stress is characterized by elevated intracellular levels of reactive oxygen species (ROS) and/or reactive nitrogen species (RNS) and is commonly observed in several renal diseases [1,2,3]. While many studies in patients with CKD and animal models of kidney injury have shown elevated levels of ROS, less is known regarding the underlying sources of increased ROS and the affected signaling mechanisms leading to kidney damage. Such knowledge would provide better opportunities to target these mechanisms at an earlier stage and possibly preventing or delaying CKD progression.

**Figure 1:**
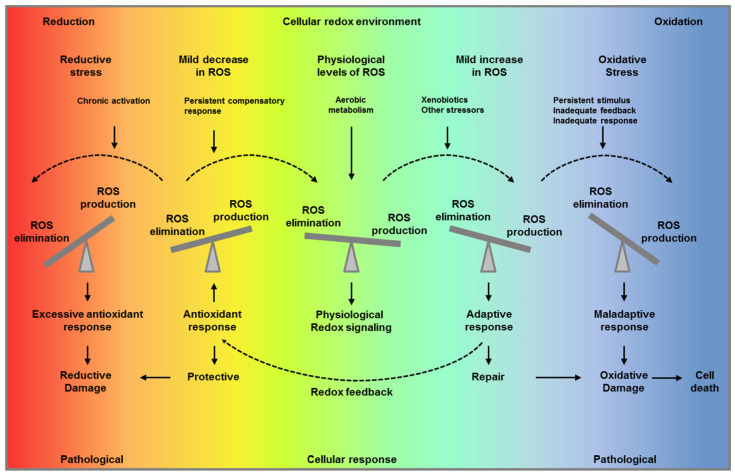
“Optimal redox environment”. Under unstressed conditions, cells maintain redox homeostasis and physiological redox signaling by metabolic fluxes and redox feedback (middle panel, green). A mild increase in reactive oxygen species (ROS) levels triggers an adaptive response to compensate and maintain ROS levels within a physiological range facilitating repair (light blue, first right panel and yellow, first left panel). When the stimulus persist overtime, and the adaptive response is inadequate or/and ROS production is overwhelming, this results in oxidative stress (blue, second right panel). On the other hand, if the compensatory response persists over time decreasing ROS levels below their physiological concentration, this impairs their signaling function and results in reductive stress (red, second left panel). Paradoxically, both reductive stress and oxidative stress are pathological conditions and can lead to ROS production and ultimate to oxidative cell damage and death.

ROS are by-products of aerobic metabolism and include the superoxide anion (O_2_^•–^), hydrogen peroxide (H_2_O_2_), and hydroxyl radicals (OH^•^). Because ROS can react with lipids, proteins, and DNA, they have been traditionally regarded as toxic molecules leading to oxidative damage. However, it is now known that ROS are also important secondary messengers in cellular signaling. Although accumulation of ROS leads to inflammation, damage, and ultimately cell death, low levels of ROS are required for pro-survival signaling, cell proliferation, growth, and energy metabolism [4,5,6]. Consequently, cells maintain tight regulation of ROS concentrations that supports normal cell function without causing cellular damage and death. Such redox homeostasis is accomplished by a strict balance between ROS production and elimination (Figure 1).

Superoxide is the product of the one-electron reduction of molecular oxygen (O_2_) and is generated by several oxidase enzymes and the mitochondrial electron transport chain (ETC). It is highly reactive and has poor diffusibility across membranes primarily being transported through anion channels [7,8]. Within the cells, is rapidly converted by superoxide dismutases 1 and 2 (SOD1 and 2) into the nonradical species H_2_O_2_, which readily crosses cell membranes and can also diffuse through aquaporin channels. In addition, H_2_O_2_ have the capacity to modify redox-sensitive proteins through the reversible thiol oxidation of reactive cysteine residues, and thereby regulate cellular function. Therefore, accumulation of O_2_^•–^ is more commonly associated with oxidative stress, whereas H_2_O_2_ is associated with redox signaling [9]. H_2_O_2_ is generated from superoxide produced by mitochondria and a family of nicotinamide adenine dinucleotide phosphate (NADPH) oxidases (NOX) [10,11]. In addition, H_2_O_2_ can react with various forms of ferrous ions, generating OH^•^. In turn, OH^•^ is extremely reactive and oxidizes indiscriminately lipids, proteins, and DNA, resulting in damage or genomic instability, which is why there exist several cellular mechanisms to maintain iron homeostasis [12]. It is worth noting that the changes in H_2_O_2_ concentration required for signaling are not associated with significant changes in the intracellular ratio of oxidized glutathione (GSSG) to reduced glutathione (GSH), or of NADPH to its oxidized form, NADP^+^ [13]. Conversely, large changes in such ratios are usually a sign of oxidative stress rather than physiological redox signaling [14].

Nitric oxide (NO), peroxynitrite (ONOO^−^), nitrotyrosine, and nitrosothiols are the principal RNS, can coexist and interact with ROS, and constitute additional players in renal pathophysiology [15]. NO is synthesized by various isoforms of nitric oxide synthase (NOS), from l-arginine, oxygen, and other cofactors, including tetrahydrobiopterin. NO can further react with O_2_^•–^ to form ONOO^−^, which can induce several protein modifications by oxidation or nitration of amino acids such as tyrosine, cysteine, methionine, and tryptophan. In addition, ONOO^−^ can interact with DNA resulting in both sugar and nucleus-base damage.

This review focuses on the main sources of ROS and antioxidant systems in the kidney, and redox signaling pathways leading to inflammation and fibrosis, which result in abnormal kidney function and CKD progression.

## 2. Main Sources of ROS in the Kidney

Several organelles including the mitochondria, endoplasmic reticulum (ER), and peroxisomes, as well as enzymatic systems such as xanthine oxidase, lipoxygenase, nitric oxide synthase, and NOX are known to be sources of ROS within mammalian cells [4]. In the kidney, mitochondria and the NOX family are the major sources of endogenous ROS [16,17,18,19].

### 2.1. Mitochondria

Kidneys require a significant amount of energy to control the body fluid composition by filtering and reabsorbing materials. Proximal and distal tubular reabsorption are primarily driven by adenosine triphosphate (ATP)-dependent active transport. Renal ATP is primarily supplied by a significant amount of mitochondria [20], double-membrane organelles that also regulate numerous cellular processes, such as cell proliferation, ROS production, calcium signaling, and apoptosis [21]. Thus, mitochondrial dysfunction can have a profound impact on renal cellular function.

Mammalian mitochondria can generate O_2_^•–^ and/or H_2_O_2_, from substrate catabolism and the electron transport chain (ETC). Aerobic respiration encompass a series of oxidation–reduction (redox) reactions, which involve the transfer of electrons between an electron donor (reducing agent) to an electron acceptor (oxidizing agent), being oxygen the final electron acceptor. Redox couples, comprising the reducing species and its corresponding oxidizing form (e.g., NADH and NAD^+^), are responsible for this electron flow, which is dependent on the state of the specific couple. During this process, electrons can leak prematurely to oxygen generating O_2_^•–^ and/or H_2_O_2_.

**Figure 2:**
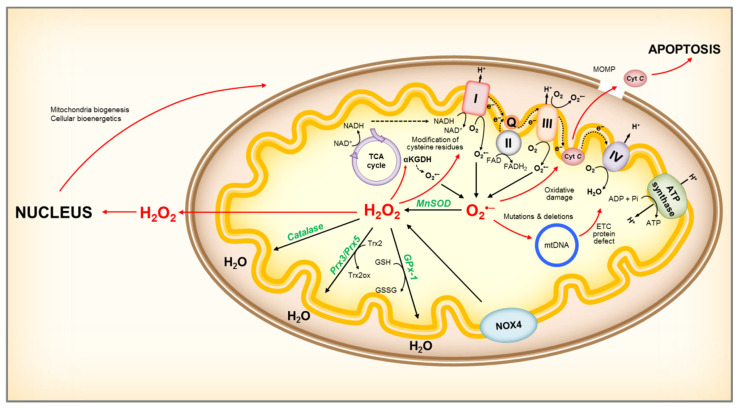
Representation of mitochondrial ROS production and its effects. NADH is oxidized to NAD^+^ by reducing FMN to FMNH_2_ in complex I, and O_2_^•–^ is produced when electrons leak from FMNH_2_, which is affected by the mitochondrial NADH/NAD+ ratio. Damage to the ETC, or accumulation of NADH due to low ATP demand and low respiration rate, will increase the NADH/NAD+ ratio and lead to O_2_^•–^ formation. In addition, complex I can produce O_2_^•–^ by reverse electron transport (RET). RET occurs when QH_2_ is oxidized to drive the reduction of NAD^+^, supported by high QH_2_/Q and high proton motive force (ΔpH) across the inner membrane. Complex III channels electrons from Q to cyt *c*, and can be an important source of O_2_^•–^ in the presence of QH_2_ and Qi site inhibitors, by allowing O_2_ to react with a semiquinone bound to the Q_o_ site. An increase in NADH/NAD+ ratio can also lead to O_2_^•–^ production at other sites connected to the NADH pool, such as αKGDH. Increases in O_2_^•–^ can lead to mitochondrial protein, DNA and membrane damage, impairing ATP production capacity, fatty acid oxidation and aa metabolism, and ultimately leading to mitochondria dysfunction. Mitochondria damage can lead to release of cyt *c* to the cytosol, activating the cell’s apoptotic machinery. NOX4 localized to the inner mitochondria membrane is an important source of H_2_O_2_. Mitochondria H_2_O_2_ can participate in redox signaling, by modulating mitochondria enzyme activity and/or acting as a retrograde signal to the cell to report on mitochondrial redox state. Within mitochondria, H_2_O_2_ can be reduced to water by catalase, GPx-1 using GSH as co-substrate, and Prx3 or Prx5 utilizing Trx2 to regenerate the active site. ROS, reactive oxygen species; O_2_^•–^, superoxide; MnSOD, manganese superoxide dismutase; H_2_O_2_, hydrogen peroxide; ETC, electron transport chain; FMN, Flavin mononucleotide; FMNH_2_, reduced FMN; Q, coenzyme Q; QH_2_, reduced coenzyme Q; cyt C, cytochrome *c*; αKGDH, alpha-ketoglutarate dehydrogenase; GSH, reduced glutathione; GSSG, oxidized glutathione; GPx-1, glutathione peroxidase-1; Prx3 or Prx5, peroxiredoxins 3 or 5; Trx2, thioredoxin 2, Trx2ox, oxidized thioredoxin 2.

Previously, ROS generation was mainly attributed to complexes I and III. However, mitochondrial proteins pyruvate dehydrogenase, α-ketoglutarate dehydrogenase, succinate dehydrogenase, have also been reported to generate mitochondria ROS (mtROS) [22]. Importantly, ROS production rate from each enzyme is highly dependent on cellular energy status, the concentration of the reducing species and hence the mitochondria redox state, in addition to the rate constant for the leak reaction [23]. Interestingly, most of these enzymes contain thiol residues adjacent to the ROS producing centers and can be modified by ROS, suggesting that redox signaling itself is a mechanism controlling mtROS production [24] (Figure 2). In addition, other factors such as mitochondria fission and fusion are likely to influence mtROS production [25]. Accordingly, changes in bioenergetic state due to e.g., caloric restriction, can influence mitochondrial substrate availability and thus result in different rates and sites of O_2_^•–^ and H_2_O_2_ production. Furthermore, mitochondria are highly dynamic organelles and can redistribute in response to cellular cues to form localized pools of mtROS that influence signaling pathways [26].

Over the past few years, increased mtROS has been implicated in numerous conditions from aging to cancer, and remains as a key mediator of kidney damage. Several studies in experimental models of kidney injury, as well as human studies have shown an increase in ROS production associated with kidney failure. Specifically, increased production of mtROS has been associated with mitochondrial dysfunction ultimately resulting in cell damage and progression of kidney disease. Consistent with these observations, the administration of mitoTEMPO, a mitochondrial targeted antioxidant, has been shown to ameliorate diabetic nephropathy [27,28]. On the other hand, in vivo determinations of mtROS utilizing dihydroethidium in diabetic mouse have been shown reduced mtROS production in diabetic environment [29]. One possible explanation for these seemingly contrasting findings could be due to a temporal effect in CKD progression, which can lead to inconclusive or contradictory reports showing both higher and lower mtROS production in the kidney. During the course of CKD, there are a series of dynamic events that may initially increase mtROS production but not able to maintain it as the disease progresses. An increase in mtROS production may damage mitochondrial DNA and ETC, ultimately leading to a reduction in mtROS [30,31].

Notwithstanding the seemingly controversies, it is well-accepted that mtROS overproduction is damaging to the cells in several ways, including nuclear and mitochondrial DNA damage and promoting cell apoptosis [32]. Yet, the classical view that only overproduction of mtROS is harmful has been recently challenged and it is now postulated that both “too much” or “too little” of mtROS may be detrimental for cell survival. Consequently, normal kidney cell function relies on the “right” amount of ROS as very low levels of mtROS may compromise several important signaling pathways and impair redox-dependent protein number, function, and activity. This dual role of mtROS may be explained if mtROS are viewed as stress-induced survival signals that serve the purpose of activating compensatory mechanisms under physiological levels. On the other hand, if the levels of ROS increase beyond a threshold or persist over time, then mtROS contribute to further mitochondrial dysfunction and cellular damage [33,34].

### 2.2. NADPH Oxidases

Seven different NOX homologues have been characterized (NOX1–NOX5, DUOX1, and DUOX2) with different activation mechanisms, heterogeneous tissue distribution and subcellular localizations. NOX4 is the principal NOX isoform in the kidney [35,36] and directly binds the integral membrane protein p22phox, which is required for NOX4 activity [36]. Unlike other NOX isoforms, its activity is regulated at the expression level due to unique conformational features of its C-terminus that facilitates spontaneous transfer of electrons from NADPH to FAD, conferring constitutive activity [37]. Another characteristic feature of NOX4 is the generation of H_2_O_2_ vs O_2_^•–^, postulated to be due to intrinsic property of its E-loop, which allows spontaneous dismutation of O_2_^•–^ [38,39].

**Figure 3:**
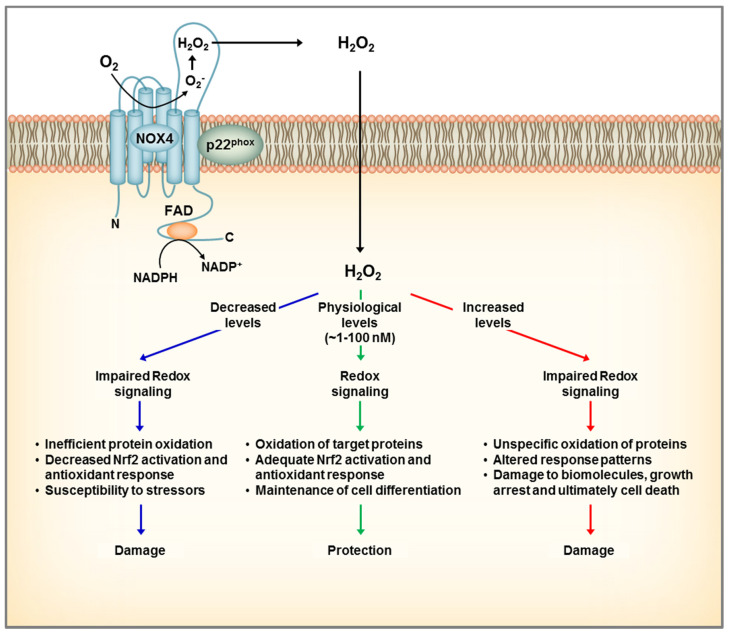
Dual role of NOX4 in the kidney. NOX4 localizes to the plasma and intracellular membranes and generates constitutively H_2_O_2_ that participates in redox signaling. At physiological conditions, NOX4 mediates a steady redox signal, which favors cellular quiescence and differentiation and overall exerting a protective role. The case of complete loss of NOX4 leads to inefficient oxidation of protein targets, which makes the cell more susceptible to challenges by stressors, and ultimately leads to cell damage. On the other hand, overexpression of NOX4 leading to increases in H_2_O_2_ levels, can result in oxidative damage to proteins and ultimately cell death.

NOX4 resides in part in the plasma membrane, but is localized predominantly to intracellular membranes in mitochondria [40], endoplasmic reticulum [41], focal adhesions [42], and nucleus [42,43]. NOX4 derived H_2_O_2_ participate in a variety of physiological functions including cell proliferation, metabolism, as well as cell death. Yet, the role of NOX4 in the kidney remains controversial. Although physiological levels of H_2_O_2_ are required for normal cell function, excessive concentrations are known to induce inflammation, fibrosis, apoptosis, and cell damage. Considering the constitutive activity of NOX4, the amount of H_2_O_2_ generated by the enzyme is dependent on the expression level. Consequently, whether NOX4 may be harmful or beneficial depends on its abundance, and the cell requirements at a given time (Figure 3).

Abnormal upregulation of renal NOX4 is proposed to play an important role in various kidney diseases including diabetic and hypertensive nephropathies, and polycystic kidney disease, by increasing ROS production and mitochondrial damage [44,45,46,47,48,49]. Importantly, there is interplay between these two ROS sources that can lead to feed-forward mechanisms where activation of one source of ROS can lead to activation of another (ROS-induced ROS production) [50]. Elevated NOX4 expression has been shown to increase mtROS production, whereas its down-regulation restores mitochondrial bioenergetics and reduces mtROS [48].

In contrast, *Nox4*^−/−^ mice subjected to unilateral urinary obstruction (UUO) injury exhibited increased interstitial fibrosis, and oxidative stress compared to WT controls. The proposed mechanisms leading to enhanced kidney fibrosis are increased tubular cell apoptosis aggravating tubular atrophy, and decreased peritubular capillaries resulting in enhanced local hypoxia. It was further speculated that the observed oxidative stress may be due to a decreased antioxidant response, suggesting a protective role under injurious conditions [51]. Indeed, NOX4 derived H_2_O_2_ have been reported to enhance the nuclear factor erythroid 2-related factor 2 (Nrf2) stability, (a main regulator of the antioxidant response), and regulate homocysteine metabolic pathway favoring GSH synthesis promoting cellular protection against oxidant insult [52,53,54]. This effect may explain why a low chronic activity of NOX4 may facilitate tissue adaptation and healing under stress conditions [55], and why NOX4 complete absence promotes kidney fibrosis and oxidative stress in a UUO model, suggesting a protective role under certain conditions [51]. In the end, it is not whether NOX4 itself is good or bad, but whether there is a pathological up or down regulation leading to abnormal concentrations on ROS.

## 3. Antioxidant Systems

There are several antioxidant systems to counteract the damaging effects of ROS and RNS in the kidney. Briefly, the antioxidant system can be subdivided into two main schemes: Enzymatic and non-enzymatic systems. The enzymatic system is comprised by SODs, catalase, glutathione peroxidase (GPx), glutathione reductase (GR), glutathione S-transferase (GST), peroxiredoxin (PRX), and thioredoxin (TRX). The non-enzymatic system is characterized by organic compounds such as ascorbic acid, α-tocopherol, carotenoids, flavonoids, and reduced GSH.

### 3.1. Superoxide Dismutase (SODs)

SODs are first in line to counteract the effects of ROS, by catalyzing the dismutation of O_2_^•–^ into O_2_ and H_2_O_2_.

2 O_2_^•–^ + 2H_3_O^+^ → O_2_ + H_2_O_2_ + 2H_2_O

There are three unique and highly compartmentalized isoforms: SOD1, or CuZn-SOD (EC 1.15.1.1), SOD2, or Mn-SOD (EC 1.15.1.1), and SOD3, or EC-SOD (EC 1.15.1.1), all of which are normally expressed in the kidney. SOD1 is found almost exclusively in intracellular cytoplasmic spaces, SOD2 in the mitochondria, and SOD3 in the extracellular spaces [56]. Most SOD activity in the mammalian kidney is through SOD1, which accounts for approximately 80%, and studies in animal models and humans have implicated reduced SOD1 activity in kidney damage [57,58,59]. Despite this, reductions in SOD2 results in a more severe pathological phenotype, highlighting the importance of mtROS. Indeed, while global SOD1 knockout (KO) mice are indistinguishable from wild type (WT) mice at birth and only display accelerated neuromuscular aging phenotypes by 5–8 months of age, complete loss of SOD2 can result in massive oxidative stress and neonatal death from cardiomyopathy, neurodegeneration, and metabolic acidosis [60,61,62].

Interestingly, a murine model developed to establish the role of mitochondrial membrane peptidase 2-like protein in the development of renal fibrosis presented significantly higher SOD-1 expression compared to age-matched controls, possibly reflecting a compensatory mechanism to overcome increased ROS production [63]. On the other hand, a murine model of DN has been reported to exhibit down-regulation of SOD1 and SOD3, but not SOD2, in the kidney as the mice aged, suggesting a more prominent role in the pathogenesis of DN [58].

### 3.2. Catalase

Catalase is a heme-containing tetramer that reduces the H_2_O_2_ generated by SOD into oxygen and water.

2H_2_O_2_ → 2H_2_O + O_2_

It is localized to peroxisomes, and highly expressed in liver, lungs, and kidneys [64]. Although catalase is highly efficient at reducing H_2_O_2_, its role may not be central in modulating H_2_O_2_ as it is mainly localized to peroxisomes. On the other hand, peroxisomes are essential organelles for lipid metabolism, generating abundant H_2_O_2_ during fatty acid (FA) β-oxidation [65]. Notably, impaired FA oxidation has been associated with CKD supporting a role for catalase in renal disease [66,67].

Catalase deficiency has been reported to contribute to increased tubulo-interstitial injury and fibrosis in a murine model of UUO, supporting the protective role in kidney injury [68]. Later studies in homozygous acatalasemic mutant mice subjected to 5/6 partial nephrectomies confirmed the role of catalase deficiency on progressive renal fibrosis by sensitizing the remnant kidney to epithelial to mesenchymal transition [69].

### 3.3. PRX, TRX, and GPX

PRX is a H_2_O_2_ scavenger, which works together with TRX to relay a series of redox reactions to reduce H_2_O_2_. In the first reaction, cysteine residues of PRX undergo oxidation by H_2_O_2_, reducing H_2_O_2_ to H_2_O. During this process, H_2_O_2_ is removed but PRX is inactivated. In the second reaction, the cysteine residues of TRX are oxidized as the inactivated PRX is reduced and reactivated. Finally, the oxidized and inactivated TRX is reduced by thioredoxin reductase, using NADPH as the reducing agent [70].

GPX is another H_2_O_2_ scavenger. Similar to the PRX and TRX, GPX and GSH collaborate to detoxify H_2_O_2_ to H_2_O, yielding an GSSG in the process, which can be subsequently reduced by glutathione reductase (GR) and NADPH [71]. In turn, NADPH is generated by the pentose phosphate pathway (PPP), one-carbon metabolism, isocitrate dehydrogenases, and malic enzymes.

There are six isoforms of PRX and eight isoforms of GPX that are extensively distributed all through the cells. Similar to SODs, PRX and GPX have distinct cellular localization, including cytosol, mitochondria, ER, peroxisomes, and extracellular space [70,72]. Overall, cells have robust antioxidant systems by which SODs convert O_2_^•–^ to H_2_O_2_, and PRXs and GPXs reduce H_2_O_2_ to H_2_O.

### 3.4. Non-Enzymatic Antioxidant Systems

In addition to the previously described enzymatic scavengers, there are other intracellular systems to counteract ROS. GSH, a Cys-containing tripeptide often found in millimolar concentrations, is the most important one. As such, the GSH pool is tightly regulated within the cells, and is associated with glucose metabolism through the PPP, which provides the NADPH required to maintain GSH in a reduced form.

GSH is synthesized from l-glutamate, l-cysteine and glycine in two steps, by γ-glutamyl-cysteine synthase and glutathione synthase, whereas redox reactions involving GSH are catalyzed by GSH peroxidases (GSH-Px) and GSSG reductases (GSSG-Rd) [73]. Determinations of total GSH and/or GSSG levels have been used to estimate the cellular redox environment, but it can be also more conveniently estimated by taking the ratio of [GSH]/[GSSG]. Importantly, intracellular GSH redox state is also known to regulate various cellular functions including gene expression, cell-cycle progression, apoptosis, and metabolism through modifications of the cellular redox environment [74,75,76].

In addition to reduced SOD and catalase levels, several rodent models of kidney disease and patients with CKD have been shown to present decreased levels of GSH early on contributing to disease progression [1,2,77,78,79,80].

## 4. Nuclear Factor Erythroid 2-Related Factor 2 (Nrf2): The Master Regulator of the Antioxidant Response

Nrf2 is a transcription factor that integrates cellular stress signals and responds by regulating the expression of several antioxidant proteins. Under basal conditions Nrf2 is kept transcriptionally inactive through binding to its main inhibitor, Kelch like-ECH-associated protein 1 (Keap-1), which targets Nrf2 for proteasomal degradation. Keap-1 contains several reactive cysteine residues that act as stressor sensors. ROS-mediated oxidation of these cysteine residues result in a conformational change of Keap-1, thereby reversing the proteasomal degradation of Nrf2, which then translocates to the nucleus [81,82,83]. In the nucleus, Nrf2 heterodimerizes with other transcription factors (e.g., small Maf) and binds to regulatory sequences (antioxidant response elements) or electrophile response elements, in the promoter regions of genes encoding antioxidant and phase 2 detoxifying molecules. In addition, phosphorylation of Nrf2 threonine or serine residues by upstream kinases such as protein kinase C (PKC), mitogen-activated protein kinases (MAPK), phosphatidylinositol-3-kinase/Akt (PI3K/AKT), casein kinase-2 (CK2), and the protein kinase RNA-like endoplasmic reticulum (PERK), results in its nuclear translocation.

Several renal diseases have been associated with an abnormal Nrf2-antioxidant response. For example, ablation of Nrf2 in experimental models is known to cause lupus-like autoimmune nephritis, and has been shown to exacerbate diabetes-induced oxidative stress, inflammation and renal damage [84,85]. Unexpectedly, experimental models of CKD associated with oxidative stress present with reduced Nrf2 activity and expression of its target gene products in kidney tissues, but the administration of the Nrf2 activator curcumin ameliorated CKD by blocking inflammatory pathways and inhibition of nuclear factor κB (NF-κB) [86,87,88,89,90]. Further, synthetic Nrf2 activators such as Bardoxolone, have demonstrated reduced glomerulosclerosis, interstitial fibrosis and inflammation through decreasing NF-κB activation in multiple rodent models of kidney disease [91,92,93,94].

These encouraging results have led to several clinical trials in patients with CKD, and type 2 diabetes (T2D) and Bardoxolone [95,96,97]. While the early phase 2 trial (BEAM) was associated with improvement in the eGFR (estimated glomerular filtration rate) that persisted over 52 weeks in patients with advanced CKD and T2D, the phase 3 trial (BEACON) was subjected to early termination due to higher rate of cardiovascular events with Bardoxolone [96]. In this study, Bardoxolone methyl did not reduce the risk of end stage kidney disease (ESRD) or death among patients with T2D and stage 4 CKD. Nevertheless, a post-hoc analysis of the BEACON trial aimed to characterize changes in kidney function showed Bardoxolone increased eGFR, which was sustained through study week 48 and 4 weeks after cessation [98]. Presently, Bardoxolone methyl is being tested in patients with CKD due to Alport syndrome (a genetic disorder characterized by progressive loss of kidney function, hearing loss, and eye abnormalities) (CARDINAL; NCT03019185), in patients with T2D and CKD in Japan (TSUBAKI; NCT02316821), and in patients with CKD 2–3 due to Autosomal Dominant PKD (FALCON; NCT03918447). CKD is complex and further studies are needed to determine whether and/or when Nrf2 activation may be beneficial to prevent or slow CKD progression.

Interestingly, the activation of the Nrf2-mediated antioxidant response confers a more reduced intracellular environment through the process of ROS detoxification [99,100]. This reduced environment is known to overall promote cell survival and proliferation and may have a detrimental effect in diseases characterized by a proliferative phenotype such as polycystic kidney disease (PKD). Intriguingly, various oxidative or electrophilic cellular stresses, including ROS and RNS, but also phenolic compounds (i.e., Nordihydroguaiaretic acid; NDGA) and some metabolites such as fumarate, modify Keap-1 cysteine residues and increase the Nrf2 antioxidant response, and have been associated with a renal cystic phenotype. For example, NDGA administration to laboratory animals induces cystogenesis and has been used as a chemical model to study PKD. Moreover, NDGA-induced cystogenesis is exacerbated by transferring germ-free rats to regular housing conditions and exposing them to environmental contaminants, which act as stressors [101,102]. Additionally, the *Fh1*^−/−^mice, a model with fumarate hydratase (FH) deficiency developed to study hereditary leiomyomatosis and renal cell cancer, has increased levels of fumarate and develops many kidney cysts [103,104,105,106].

## 5. Mechanisms of Redox Signaling

O_2_^•–^ and H_2_O_2_ are signaling molecules acting on downstream pathways, such as transcription factors, tyrosine kinases/phosphatases, ion channels, mitogenic factors, and cytokines. By acting through these signaling pathways, ROS have the capacity to influence several kidney functions such as tubular sodium transport, tubuloglomerular feedback and medullary blood flow. But in addition, ROS molecules can control cell migration and growth, inflammatory responses and extracellular matrix deposition, and apoptosis. Ultimately, the effect of ROS within different components of the kidney depends on the local concentration of ROS, determined by the balance between production and antioxidant response.

H_2_O_2_ mediated oxidation of cysteine residues within proteins is one of the main mechanisms involved in redox signaling [107]. At physiological pH, cysteine residues exist as a thiolate anion (Cys–S-), which are more prone to oxidation compared with the protonated cysteine thiol (Cys-SH) [108]. The oxidation of the thiolate anion to the sulfenic form (Cys–SOH) by nanomolar concentrations H_2_O_2_ causes reversible allosteric changes within the protein, resulting in changes to protein function. The reduction of the sulfenic form by disulfide reductases TRX and GRX to thiolate anions returns the protein to its original state and function [109]. Interestingly, higher concentrations of H_2_O_2_ are estimated to further oxidize thiolate anions to sulfinic (SO_2_H) or sulfonic (SO_3_H) species. However, contrary to sulfenic modifications, sulfinic and sulfonic modifications can be irreversible and result in permanent protein damage, which is why cells are equipped with antioxidant systems. Cells therefore have professional enzymes dedicated to prevent the buildup of intracellular H_2_O_2_—primarily, PRX and GPx.

## 6. ROS in Inflammation and Fibrosis

Increases in ROS in tubular epithelial cells leads to the release of pro-inflammatory cytokines and chemokines as a mechanism for repair. However, uncontrolled or persistent increases in ROS result in inflammation and fibrosis leading to renal damage and CKD progression.

**Figure 4:**
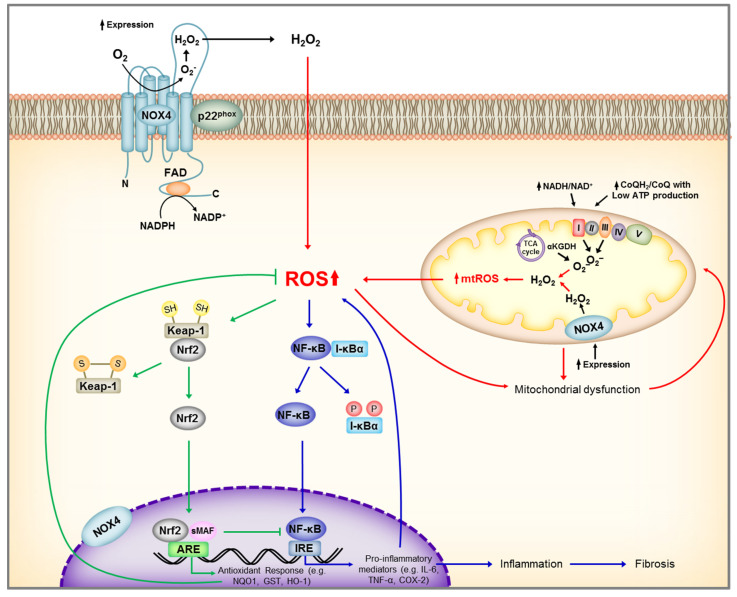
Schematic representation of main renal sources of reactive oxygen species (ROS) and Nrf2, NF-KB activation by ROS. Under unstressed conditions, Nrf2 is maintained in the cytoplasm through its repressor molecule, Keap1. ROS, as well as electrophilic and phenolic compounds, modify Keap-1 cysteine residues resulting in dissociation of Nrf2-Keap1 complex, and Nrf2 nuclear translocation. In the nucleus, Nrf2 dimerizes with other transcription factors, such as small Maf, and promotes transcriptional activation of antioxidant and detoxifying enzymes by binding to the antioxidant responsive elements (ARE) in the promoter regions of the target genes. At the same time, ROS activates IκB kinase (IKK) leading to phosphorylation of the repressor molecule I-κB, and translocation of NF-κB to the nucleus leading to transcriptional activation of genes encoding inflammatory cytokine and chemokines. ROS, reactive oxygen species; NADP+, Nicotinamide adenine dinucleotide phosphate; NADPH, reduced form of NADP^+^; NOX4, NADPH Oxidase 4; FAD, flavin adenine dinucleotide; Keap-1, Kelch-like ECH-associated protein 1, Nrf2, nuclear factor erythroid 2–related factor 2; ARE, antioxidant response element; NQO1, NAD(P)H Quinone Dehydrogenase 1; GST, Glutathione S-transferase; HO-1, Heme oxygenase-1; sMAF, small MAF SH, protein thiol of cysteine; S-S, disulfide bond; NF-κB, Nuclear Factor kappa-light-chain-enhancer of activated B cells; P, phosphorylation; TNF-α, Tumor Necrosis Factor alpha; COX-2, cyclooxygenase-2.

NF-κB consists of a family of transcription factors that play critical roles in inflammation, as well as cell proliferation, differentiation, and survival [110]. Several in vitro studies, as well as experimental animal models and human studies have shown persistent activation of NF-κB and its pathogenic role in mediating chronic inflammation in CKD [111,112,113,114]. This redox-sensitive transcription factor can modulate DNA-binding activity of its multiple homo- and heterodimers, activate or inactivate the inhibitory κB kinase complex, or activate NF-κB by alternative inhibitor κBα phosphorylation [115]. Furthermore, the existence of pro-oxidant and antioxidant NF-κB targets shows the degree of complexity between ROS and NF-κB signaling (Figure 4).

The ultimate consequence of persistent inflammation and consequent accumulation of extracellular matrix is renal fibrosis, which is a common characteristic of CKD. Mesangial and fibroblast activation have been identified as major effectors in extracellular matrix deposition through several cellular pathways. Transforming growth factor-beta (TGF-β) is known to have a critical role [116]. ROS upregulation of TGF-β in mesangial and endothelial cells is known to stimulates SMAD signaling enhancing the expression of collagen 1, 3, and 4, fibronectin, and plasminogen activator inhibitor-1, while attenuating activity of extracellular matrix (ECM) degradation factors [117,118,119,120]. In line with this, previous studies have shown that NOX4 plays a critical role in TGF-β mediated fibrosis in various organs, including the kidney, positioning pharmacological inhibition of NOX4 as an attractive therapeutic approach in CKD [121,122].

## 7. Concluding Remarks

To date, substantial evidence indicates that altered ROS-antioxidant systems play a major role in chronic kidney diseases. However, since ROS molecules are also part of physiological processes, it is difficult to precisely define clear pathogenic mechanisms. Furthermore, it is possible that ROS-antioxidant levels fluctuate during the variable course of many kidney diseases. Therefore, to achieve therapeutic benefits with minimum adverse effects, it is crucial to target ROS at the time and/or cellular location that selectively benefits diseased cells. Importantly, the dosage of the anti- and/or pro-oxidant must be regulated to preserve the redox signaling necessary for the healthy cells. Similarly, anti- and/or pro-oxidants must be administered at specific timing that has become dysregulated and at a dose that does not impede normal responses. Therefore, significant medical advances could arise from an improved understanding of redox regulation with high temporal, spatial, and quantitative resolution.
